# β-Thalassemia and ocular implications: a systematic review

**DOI:** 10.1186/s12886-016-0285-2

**Published:** 2016-07-08

**Authors:** Aliki Liaska, Petros Petrou, Constantinos D. Georgakopoulos, Ramza Diamanti, Dimitris Papaconstantinou, Menelaos G. Kanakis, Ilias Georgalas

**Affiliations:** 1st Department of Ophthalmology, Athens University, Mesogeion 154, Athens, 11527 Greece

## Abstract

**Background:**

Beta-thalassemia is a severe genetic blood disorder caused by a mutation in the gene encoding for the beta chains of hemoglobin. Individuals with beta-thalassemia major require regular lifelong Red Blood Cell transfusions to survive. Ocular involvement is quite common and may have serious implications.

**Methods:**

Extensive review of observational studies on beta-thalassemia, to determine the prevalence and spectrum of ocular abnormalities, by clinical examination and multimodal imaging, and to investigate risk factors for their development.

**Results:**

Frequency of ocular involvement differs among various studies (41.3–85 %, three studies). Ocular findings in beta-thalassemia may correlate to the disease itself, iron overload or the chelating agents used. Beta-thalassemia ocular manifestations include ocular surface disease, as demonstrated by tear function parameters (two studies). Lens opacities are present in 9.3–44 % (five studies). Lenticular opacities and RPE degeneration correlated positively with use of desferrioxamine and deferriprone respectively (two studies). Ocular fundus abnormalities characteristic of pseudoxanthoma elasticum (PXE), including peau d’orange, angioid streaks, pattern dystrophy-like changes, and optic disc drusen are a consistent finding in seven studies. Patients with PXE-like fundus changes were older than patients without these fundus changes (two studies). Age (two studies) and splenectomy (one study) had the strongest association with presence of PXE-like fundus changes. Increased retinal vascular tortuosity independently of the PXE-like fundus changes was found in 11–17.9 % (three studies), which was associated with aspartate amino transferase, hemoglobin and ferritin levels (two studies). Fundus autofluorescence and electrophysiological testing (ERG and EOG) may indicate initial stages or more widespread injury than is suggested by fundus examination (two studies).

**Conclusions:**

Beta-thalassemia may present with various signs, both structural and functional. Pseudoxanthoma elasticum like fundus changes are a frequent finding in patients with b-thalassemia. These changes increase with duration or severity of the disease. Retinal vascular tortuosity may be an additional disease manifestation related to the severity and duration of anemia and independent of the PXE-like syndrome. Patients with long-standing disease need regular ophthalmic checkups because they are at risk of developing PXE-like fundus changes and potentially of subsequent choroidal neovascularization.

## Background

Thalassemia is a severe genetic blood disorder caused by a mutation in the globin gene. Abnormal globin chains lead to the excessive destruction of red blood cells [1]. The phenotypes of homozygous or genetic heterozygous compound beta-thalassemias include thalassemia major (TM) and thalassemia intermedia (TI). Individuals with thalassemia major usually come to medical attention within the first two years of life. These patients require lifelong RBC transfusions at regular intervals to survive. Thalassemia intermedia includes patients with milder symptoms, who present at an older age and do not require regular transfusions [2]. More than 42,000 newborns are affected by Beta-thalassemia every year worldwide. Without blood transfusions, Beta-thalassemia major (TM) causes death amongst infected children before the age of 3 years old [3]. Although transfusions can prevent death and decrease mortality, iron accumulated from transfused red blood cells can lead to organ failure [4, 5]. Iron chelation treatment, to reduce iron store in the body and improve the long-term survival rate of patients with TM, is considered a mandatory adjuvant therapy.

As a group, the thalassemias are the most common single gene disorder in the world. High prevalence occurs in developing regions as well as in large multiethnic Western cities due to an expanding immigrant population [6]. The inheritance of β-thalassemias is recessive. The mutations in the β-globin gene and consequent defective β-chain production leads to a devastating cascade: imbalance in α/β- globin chain synthesis, ineffective erythropoiesis, reduced red blood cell survival and subsequent anemia [7]. Although the disease is confirmed genetically, the phenotype of β-thalassemia is determined based on clinical observation.

Therapeutic measures resulted in a progressive improvement in life expectancy in both developed and developing countries [8–16]. Increased awareness, better education and optimal health care provision efforts, a large body of evidence attained by clinical trials and observational studies conducted in the last 3 decades, allowed for remarkable advances in diagnostic and therapeutic options. Milestones in this effort include the introduction of guidelines for safe processing of blood products, noninvasive techniques for the assessment of iron overload in target organs, oral iron chelators, and prevention/management schemes for specific complications [17].

Thalassemia major is a multidimensional medical, social, and psychological problem. The course of thalassemia patients depends on the availability of adequate blood transfusion and other therapeutic modalities. The closer and more systematic follow-up of thalassemia patients, along with the significant improvement of available treatments, prolonged life expectancy lead to the gradual broadening of the clinical spectrum of beta thalassemia with new, previously unknown manifestations [18].

The purpose of the present review is to identify the whole spectrum of ocular complications of thalassemia presented in the literature and provide an extensive review on both functional and structural abnormalities related to chelation therapy. We also underline the need for updated guidelines for screening and follow up of thalassemia patients and the proper utilization of multimodal imaging techniques.

## Methods

### Study sources and searches

This is a systematic review of the literature regarding the ocular implications of β-Thalassemia. The literature search was conducted in Medline, PubMed, Embase, ISI website of knowledge and The CENTRAL of Cochrane Library, to identify relevant published in English and German articles, up to January 22^nd^ 2016 (consecutive search - final search update). The search key words and subject terms were used included “thalassemia AND ocular findings” “deferoxamine AND ocular toxicity” “deferiprone AND thalassemia AND retina” “deferasirox AND thalassemia AND retina” “iron chelators AND Thalassemia AND retina”. Relevant articles in reference lists of published articles were also searched. We collected and retrieved a total of 126 publications. From the 126 publications, we included a total of 81 references.

In Pub Med, we used the advanced search mode, to build our search, by using the keywords: “thalassemia, ocular findings, deferoxamine, ocular toxicity, deferiprone deferasirox, iron chelators, retina”. Then, we combined them in the builder to find the most high – yielding and relevant combinations. By employing the above strategy we defined the search terms as described above (“thalassemia AND ocular findings” etc.). The same terms were used to feed the search engines for all the Databases.

In Pub Med the search yielded the following results:

- “thalassemia AND ocular findings”: 49 results
- “deferoxamine AND ocular toxicity”: 48 results
- “deferiprone AND thalassemia AND retina”: one results
- “deferasirox AND thalassemia AND retina”: two results
- “iron chelators AND thalassemia AND retina”: five results

We also expanded our search to similar articles, as suggested by the search engine. On line first articles were included.

### Study selection and data extraction

All studies that were identified by the literature searches were reviewed and selected according to the following prior criteria: (i) patients with thalassemia regardless of severity, age and sex; (ii) ocular findings (iii) case-control studies on deferoxamine ocular toxicity (iv) case reports were included only if they were sufficiently documented and provided novel data. Full text articles were obtained. Data were independently extracted from each article, evaluated and further processed.

## Results

Below we present the findings of case control and case series studies regarding ocular findings in β-thalassemia.

Jafari et al. [19], in a cross sectional, controlled study, examined 54 thalassemia major patients. All the thalassemic patients were asymptomatic. Ocular findings including dry eye (33.3 %), cataract (10.2 %), retinal pigment epithelium degeneration (16.7 %), color vision deficiency (3.7 %), and visual field defects (33.7 %) were detected in 68.5 % of thalassemic group. The prevalence of ocular abnormalities in the control group of normal individuals was 19.4 %, significantly lower than that in thalassemia patients (*P* = 0.000). The was no statistically significant correlation between ocular abnormalities and mean serum ferritin level (*P* = 0.627) and mean hemoglobin concentration (*P* = 0.143). Finally, the number of blood transfusions was positively correlated with the presence of ocular abnormalities (*P* = 0.005).

Barteselli et al. [20] in a cross-sectional, observational study involving a total of 255 patients with β-thalassemia major (TM: 153 patients) and β-thalassemia intermedia (TI: 102 patients) report ocular fundus abnormalities characteristic of pseudoxanthoma elasticum (PXE) detected by cSLO in 70 of 255 patients (27.8 %). These included peau d’orange (19.6 %), angioid streaks (12.9 %), pattern dystrophy-like changes (7.5 %), and optic disc drusen (2.0 %). Pseudoxanthoma elasticum-like (PXE-like) changes were more frequent in patients with TI (*P* <0.001). Patients with PXE-like fundus changes were older than patients without these fundus changes (*P* <0.001). In both patients with TI and TM, age (*P* = 0.001) and splenectomy (*P* = 0.001) had the strongest association with presence of PXE-like fundus changes in multivariate analyses. A total of 43 of 255 patients (16.9 %) showed increased retinal vascular tortuosity independently of the PXE-like fundus changes, which was associated with aspartate amino transferase (*P* = 0.036), hemoglobin (*P* = 0.008), and ferritin levels (*P* = 0.005). The authors concluded that PXE-like fundus changes are a frequent finding in patients with β-thalassemia. In TI, these changes increase with duration or severity of the disease. This particular ocular phenotype suggests an ocular pathology similar to PXE. Retinal vascular tortuosity may be an additional disease manifestation independent of the PXE-like syndrome. Patients with long-standing disease requiring iron-chelating treatment and those with a history of splenectomy need regular ophthalmic checkups because they are at risk of developing PXE-like fundus changes and potentially of subsequent choroidal neovascularization.

In a controlled study conducted by Nowroozzadeh et al. [21] in 47 patients (94 eyes) to investigate the effect of altered bony orbit in beta thalassemia major on ocular biometry found that shorter axial length, thicker lens, steeper corneal curvature and more against-the-rule pattern were common findings in patients with thalassemia major.

In a prospective (1 year follow-up) observational study by Taneja et al. [22], 45 beta-thalassemia major children under regular transfusion therapy aged between six months and 21 years,assigned in groups according to the thalassemia treatment regimes they followed at the time of presentation. Group A received only blood transfusions (six patients), Group B blood transfusions with subcutaneous desferrioxamine (six patients), Group C blood transfusions with desferrioxamine and oral deferriprone (13 patients) and Group D blood transfusions with deferriprone (20 patients). Ocular involvement was detected in 26/45 patients. Also, 18/45 patients had lenticular opacities. None of these opacities was located in the visual axis and therefore none of them interfered with vision. Lens opacities correlated significantly with higher average serum iron levels, ferritin levels and number of blood transfusions received (*P* <0.001). Unaided visual acuity was found normal in 30/45 patients. Retinal Pigment Epithelium (RPE) degeneration was found in 15/45 patients and RPE mottling was seen in 4/45 patients, both more common with increasing age. Statistical significance was found between RPE degeneration or RPE mottling and higher average serum iron levels, serum ferritin levels and number of blood transfusions received but not the iron-chelating agent used. The same was true for venous tortuosity. The authors admit that the study cannot provide conclusive evident regarding the cardinal question: Are ocular changes a result of the disease per se or they emerge due to therapy with iron-chelating agents.

In a cross-sectional study, Jethani et al. [23] studied 112 children with beta-thalassemia (224 eyes), aged 4–15 years, of whom 95 children (190 eyes, 84.8 %) not on desferrioxamine therapy (case group) and 17 children (34 eyes, 15.2 %) on desferrioxamine (control group). They found that conjunctival blanching and isolated cataractous changes in the lens were the most common anterior segment findings in the untreated children (case group). Both conditions coexisted in 12 eyes. In the control group, although no lens opacities had been detected, 4/17 children had tessellated fundus. This suggests that serum ferritin levels and iron load may not be the direct cause for the beta thalassemia ocular manifestations. According to authors, this study excludes desferrioxamine of any causal role in ocular surface disease in thalassemia patients. Rather, it is thalassemia itself the most probable cause of these disorders.

Gartaganis et al. [24] in a controlled study of 52 beta-thalassemia patients (104 eyes) examined tear function parameters and conjuctival changes and reported that in ocular surface disorder of beta-thalassemia patients goblet cell loss and conjunctival squamous metaplasia was a constant finding.

Jiang et al. [25] undertook an electroretinographic (ERG) controlled study on 11 patients with beta-thalassemia major to test the hypothesis that scotopic retinal function is altered in transfused thalassemia patients on chronic Deferoxamine (DFO). The authors came to the conclusion that the gradual accumulation of iron, rather than DFO toxicity underlies the scotopic dysfunction detected in older thalassemia patients, some of whom may have had extended periods of transfusion without the protection of chelation. Thus, monitoring of retinal function is recommended in such patients.

In a prospective non-controlled cohort study conducted by Dennerlein et al. [26] to evaluate the presence of ocular side effects related to Desferrioxamine (DFO) treatment, examined 17 patients on DFO treatment: lens opacities were found in 41 % (7/17), changes in the retinal pigment epithelium in 35 % (6/17), tortuosity of retinal vessels in 24 % (4/17), dilation and sheathing of the retinal vessels in 18 % (3/17), defects in color vision in 29 % (5/17), and abnormal dark adaptation in 18 % (3/17) of the patients. The authors concluded that the ocular toxicity of DFO is dose-dependent. Major side effects including depression of the visual acuity are partially reversible by discontinuing the therapy. Under this light, regular ophthalmic evaluation becomes a necessity. Sorcinelli et al. [27] in a case series of 53 patients with Cooley’s disease in treatment with transfusions and desferrioxamine in subcutaneous infusion, report fundus mottling appearance like “leopard skin” (15 %) as the most frequent ocular change. Additional findings were lens opacity (11 %), drusen (7 %), retinal venous tortuosity (5 %), without impairment of visual acuity. According to the authors, the pathogenic factors of the ocular change are related to abnormality of iron metabolism and these results suggest that the involvement of desferrioxamine to remove iron from the eyeball is relatively small.

Gartaganis et al. [28] examined 29 patients with homozygous beta thalassemia and found that 12/29 patients had one or more ocular abnormalities. 5/29 patients with degeneration of the retinal pigment epithelium, 1/29 with lens opacities, 2/29 with lens opacities and degeneration of the retinal pigment epithelium, 1/29 with vascular abnormalities and degeneration of the retinal pigment epithelium, 1/29 with angioid streaks, lens opacities, and degeneration of the retinal pigment epithelium, and 2/29 with angioid streaks and degeneration of the retinal pigment epithelium. These abnormalities were not restricted in the thalassemia major group, as patients with thalassemia intermedia presented with the same findings. As expected, the frequency of these ocular abnormalities increased with age. There was no correlation between the abnormalities observed and the serum ferritin level, the mean hematocrit value, and the dose of desferrioxamine, given to the patients.

De Virgiliis et al. [29] studied 15 children, (9 to 16 years old), with transfusion dependent thalassemia major and moderate iron overload (serum ferritin concentrations between 1100 and 2000 μg/l). They were treated with a combination of daily subcutaneous infusion and monthly intravenous administration of very large doses od desferrioxamine (according to a previously published scheme), a result of inadequate compliance with the usual daily infusion scheme. This study provided evidence that high doses of intravenous desferrioxamine infused over a short period may lead to a reversible minor toxic effect on the retina, characterized by reduced amplitude on adapted electroretinography and defective dark adaptation. The severity of toxicity on retina and optic nerve with symptoms as night blindness, field defects, visual loss, loss of color vision, and delayed visual evoked potential was correlated to intravenous or subcutaneous infusion of similar large doses of desferrioxamine for a prolonged period. Longitudinal studies in some of these patients revealed a trend towards the evolution of pigmentary degeneration of the retina indicating toxicity at the level of retinal pigment epithelium. The depletion of substances such as iron, zinc, and copper may be related to these findings.

Gelmi et al. [30] conducted an electroretinographic and visual-evoked potential (VEP) study in 31 thalassemic patients who had never received high doses of DFO. The abnormalities found were very similar to those reported in early siderosis bulbi and included a b1-wave of significantly higher amplitude at 1 min and at the alpha point. Also, VEPs showed a N1-P1 amplitude significantly greater than in controls. According to the authors, these findings, which were more marked in older patients, point to an important causative role of iron in their genesis.

In a small case series, Davies et al. [31] followed up four beta thalassemia major patients on intravenous DFO therapy at higher doses than previously reported. Two of them developed retinal abnormalities with symptoms as night blindness and field defects. Both showed improvement on discontinuation of the drug. One of the two affected patients died in the course of the study from heart failure, and Rahi et al. [32] were able to obtain and examine an eye. They reported a complete set of histochemical, light microscopic, scanning and transmission electron microscopic findings. It was the first report that documented light and electron microscopical changes in the retinal pigment epithelium (RPE) following treatment with high dose desferrioxamine for systemic iron overload. The changes described include loss of microvilli from the apical surface, patchy depigmentation, vacuolation of the cytoplasm, disorganization of the plasma membrane, swelling and calcification of mitochondria. In addition, Bruch’s membrane overlying degenerated RPE cells appeared abnormally thickened owing to the accumulation of large amounts of mature elastic fibres, pre-elastic oxytalan, and long spacing collagen.

Haimovici et al. [33] in a well characterized case series studied 16 patients with desferrioxamine-induced retinal toxicity, described some early and unusual features. The authors assessed the role of diagnostic tests in the diagnosis and management of patients with the disorder and confirmed previously reported findings in patients with established disease, including pigmentary changes in the macula and/or the peripheral fundus, reduced amplitudes in the electroretinography (ERG), and reduced electrooculographic (EOG) light-peak to dark-trough ratios. Peripapillary, papillomacular, and paramacular patterns of retinal pigment epithelial (RPE) degeneration were each observed in one patient. Diffuse RPE or outer retinal fluorescence by fluorescein angiography was a marker for active retinopathy (both at the onset of disease and during recurrence) and preceded the development of RPE pigment mottling. The authors concluded that unusual patterns of desferrioxamine retinopathy may occur in addition to the foveomacular and/or peripheral patterns previously described. Fluorescein angiography is particularly useful for determining whether there is ongoing retinal/RPE injury. ERG and EOG testing may indicate early onset or extended widespread injury, more severe than suggested by funduscopy. Patients who do not discontinue desferrioxamine after the development of retinopathy risk further retinal/RPE injury and visual deterioration

Taher et al. [34] in a cross-sectional study of 84 randomly selected thalassemia patients reported that visual acuity (VA) was affected in 13 patients and changes in the retinal pigment epithelium were detected in 21 of them. Decreased visual acuity was significantly associated with the type of thalassemia (*P* <0.05) and a history of splenectomy (*P* = 0.05). Increased retinal vascular tortuosity was present in 14 patients. Changes regarding the retinal pigment epithelium, were significantly correlated to the type of iron chelation: patients on Deferriprone were more likely to have RPE degenerations as compared to patients on Desferrioxamine.

Viola et al. [35] described macular lesions in 40 eyes of 20 beta thalassemia patients with desferrioxamine (DFO) retinopathy (minimum duration of DFO treatment 10 years), and documented their course using multimodal imaging in a retrospective chart. Imaging included fundus photography, near-infrared reflectance and fundus autofluorescence on confocal laser scanning ophthalmoscope (cSLO),and spectral domain optical coherence tomography. Ten patients (50 %) showed a variety of pattern dystrophy-like fundus changes, including butterfly shaped-like (*n* = 3), fundus flavimaculatus-like (*n* = 3), fundus pulverulentus-like (*n* = 3), and vitelliform-like (*n* = 1) changes. Ten patients (50 %) presented with negligible changes in the macula; these patients were significantly younger than patients presenting other patterns (*P* = 0.023). Confocal laser scanning ophthalmoscope and spectral domain optical coherence tomography revealed a wide diversity in these abnormalities. They also seemed to be more widespread than suggested by slit lamp biomicroscopy alone. Abnormal fundus autofluorescence and/or near-infrared reflectance are produced by the accumulation of material within the outer retina or in the Bruch membrane-retinal pigment epithelium (RPE) complex. Follow-up examinations during a 40-month period revealed progressive development of RPE atrophy in areas of pattern dystrophy-like changes. This could be attributed to the fact that DFO retinopathy included a variety of pattern dystrophy-like changes or minimal changes affecting the RPE-Bruch membrane-photoreceptor complex. Multimodal imaging revealed the broad spectrum and real incidence of fundus changes in beta thalassemia patients, also confirming the evidence of previous histologic description of DFO retinopathy, suggesting that photoreceptor outer-derived retinoids, various fluorophores, and RPE displacement or clumping are involved in DFO retinopathy. These changes finally induce the RPE atrophy in most cases of pattern dystrophy-like changes.

Viola et al. [36] conducted a prospective, cross-sectional, case-control study on 197 consecutive patients with β-thalassemia major or intermedia with at least 10 years of treatment with DFO (79 thalassemic patients without a history of chelation therapy were included as a control group) observed abnormal Fundus Autofluorescence (FAF) not related to other diseases in 18 of the 197 patients (9 %) and was classified into 4 phenotypic patterns: minimal change, focal, patchy, and speckled. The abnormal increased or decreased FAF was bilateral in all the cases, and only in some cases did it correspond to funduscopically visible alterations. There were no FAF abnormalities in the control group. During the follow-up, progressive FAF changes related to retinal pigment epithelium (RPE) damage occurred in the patchy pattern, associated with decreasing Best Corrected Visual Acuity (BCVA). Patients with speckled and focal patterns showed limited or no changes in FAF during the follow-up. No changes in FAF were found in patients with a minimal change pattern. No treated patient with a normal baseline examination demonstrated FAF changes. Patients with patterns other than the minimal change showed significant BCVA deterioration (*P* <0.001). The authors concluded that various phenotypic patterns of abnormal FAF can be identified with cSLO imaging. Fundus autofluorescence is a helpful, fast, and noninvasive tool that allows close monitoring of the macula in patients at risk of DFO retinopathy. It may be useful in the decision to discontinue or switch the therapy in cases of particular high risk for disease progression. The progressive changes at the level of the RPE may play an important role in the evolution of visual loss during long-term treatment with DFO.

Aksoy et al. [37] report mean peripapillary Retinal Nerve Fiber Layer significantly thinner in all four quadrants in the thalassemia major group (47 patients) versus iron deficiency anemia (IDA), group (IDA: 22 patients) and healthy controls (35 individuals) (*p* <0.01), and in only the inferior quadrant in the IDA group (*p* <0.05). They also documented a positive correlation between average RNLF thickness and mean hemoglobin concentration (*r* = 0.488; *p* <0.001) and a negative correlation with the mean ferritin level (*r* = −0.544; *p* <0.001). Finally, no correlation was found between average RNLF thickness and the mean number of transfusions or the mean visual acuity of these patients (*p* >0.05).

Incorvaia et al. [38] conducted a retrospective matched controlled study to investigate retinal venous tortuosity (RVT) and possible associations with other disease parameters in 36 patients with beta-thalassemia and found significantly greater mean venous length in the thalassaemic group which was significantly associated to patient’s age. The authors concluded that patients with beta-thalassemia major have increased RVT, as compared to normal subjects. Given that in this selected population, patient’s age, (closely related to anemia duration) is the only variable responsible for the RVT increment. This clinical sign may suggest a long-standing duration of anemia. Aksoy et al. [39] in an age/sex matched controlled cross-sectional study investigated ophthalmic findings in 43 children with thalassemia major and found that In TM, Schirmer test scores are less than normal, while corneal thickness, axial length, and tear break-up time (BUT) are close to controls.

Several case reports on β-thalassemia have been retrieved in our bibliography search. These reports include: high-dose intravenous Desferrioxamine-related ocular toxicity [40] which partially recovered following cessation of DFO, macular vitelliform lesion in desferrioxamine-related retinopathy [41], loss of vision associated with angioid streaks in beta-thalassemia intermedia [42], rapidly progressing bilateral cataracts in a patient with beta thalassemia and pellagra [43] and Takayasu’s arteritis presenting with temporary loss of vision in a 23-year-old woman with beta thalassemia trait [44].

## Discussion

Patients with beta-thalassemia may present with various ocular signs both structural and functional. Frequency of ocular involvement differs among studies: (Gartaganis et al. [45], reported figures of 41.3 %, Jafari et al. reported 68.5 %, Taneja et al. [22], reported figures of 58 %, Abdel-Malak [46] reported 85 %). Ocular findings in beta-thalassemia may correlate to the disease itself, iron overload or the chelating agents used. The patient’s environmental and socioeconomic status, is a major determinant of life span and the occurrence of systemic symptoms [7]. Therefore, variety of the symptoms may be attributed to differences by regions. The course of the disease in patients with TM, subjected to regular blood transfusions, and chelation therapy may affect the spectrum of systemic symptoms [47].

### Ocular surface disease

Ocular surface disease as demonstrated by alterations in tear function parameters (reduced BUT and increased Rose Bengal staining [19, 24] and reduced Schirmer test values [24, 39], has been attributed to goblet cell loss as well as squamous metaplasia of the conjunctiva [24]. Possible explanations include trace elements and vitamin deficiencies, peroxidative damage caused by either vitamin E deficiency or environmental UV radiation. UV radiation leads to the formation of intracellular peroxide in cultured epithelial cells [48]. Thalassemia patients seem to be more vulnerable to UV radiation through peroxidative tissue injury because of secondary iron overload caused by lifelong blood transfusion [49]. Vitamin E is regarded a highly efficient antioxidant. The low vitamin E plasma levels reported in beta-thalassemia patients may also affect the oxidant/antioxidant balance and render the ocular surface more susceptible to in vitro oxidative modification [50]. In a study conducted on rats with iron overload, hemosiderin deposits were detected in macrophages mainly located in the connective tissue of lacrimal glands [51]. This may explain the impaired Schirmer test as an indicator of decreased tear production.

### Refractive status

Refractive status differences between TM patients and healthy controls are not consistent among studies. Nowroozzadeh [21] reported that children with TM had higher percentage in terms of against the rule astigmatism (typically corneal and lenticular astigmatism) and shorter axial length but this was not confirmed by Aksoy [39]. In the study by Jafari et al. [19], although no significant difference was found between spherical equivalent, the mean ± SD values for uncorrected visual acuity was 0.93 ± 0.14 in thalassemia patients, significantly different from that in normal group (0.84 ± 0.27, *P* = 0.016). However, corrective lens normalized visual acuity in all subjects of both groups.

### Anterior segment abnormalities

Anterior segment abnormalities consist mainly of lens opacities. In relevant studies, this ratio was reported as high as 4/43 (9.3 %) by Aksoy [39], 44 % byTaneja [22], 41 % by Dennerlein [26], 8/80 (10 %) by Abdel-Malak [46],10.2 % by Jafari [19] and 11 % by Sorcinelli [27]. Lens opacities are one of the most important factors for the reduction of visual acuity in children with TM and may interfere with vision if they are near the visual axis [22, 39]. Significant correlation of lens opacities with higher average serum iron levels, ferritin levels and number of blood transfusions received (*P* <0.001), has been reported by Taneja [22]. Iron-chelating agents have also been implicated as causative agents of lens opacities, although it has not yet been established a correlation between occurrence of lens opacities and dose of desferrioxamine received [46, 52]. Oxidative damage of the lens by iron overload or disturbance of oxidant/antioxidant balance may be a causative factor of lens opacities in beta-thalassemia patients [22, 43].

### Fundus changes

Fundus changes in b-thalassemia syndromes are frequent, age-dependent, and similar to those reported for PXE except for the presence of retinal vascular tortuosity. Peau d’orange was most frequent, followed by angioid streaks and pattern dystrophy-like changes, which is in line with the suggested by Barteselli sequence of occurrence in the natural disease course in PXE [20]. Beta-thalassemia intermedia carries a higher risk of developing these abnormalities than TM, especially in cases of previous splenectomy and in cases that needed transfusions and treatment with iron-chelating agents. Because these changes, in particular angioid streaks and pattern dystrophy-like changes, are well known to predispose to sight-threatening complications such as Choroidal Neovascularization (CNV), regular ocular checkups are essential for patients with b-thalassemia, in particular if elderly, splenectomized, and affected by severe TI requiring lifelong blood transfusions and consequent iron chelation therapy [20].

### PXE-like fundus changes

PXE-like fundus changes are a consistent finding in numerous studies. The term PXE-like syndrome has been used to describe vascular, dermal and ocular alterations characteristic of PXE that occur secondary to other diseases or due to genetic mutations different from those in PXE. These include, among others, hemoglobinopathies, such as beta-thalassemia or sickle-cell disease [18]. PXE-associated fundus changes consist of peau d’orange encompassing the circular periphery, comet tail lesions within the periphery, angioid streaks not exceeding peau d’orange and central pattern dystrophy-like changes. Retinal alterations characteristic of PXE are well described in these patients except comet tail lesion within the periphery. Optic disc drusen are not pathognomonic but a frequent finding. Retinal involvement increases with age [53]. Peau d’orange may represent the first retinal sign of the progressive calcification of Bruch’s membrane in beta-thalassemia. Subsequently, the extensive calcification of the Bruch’s membrane is supposed to lead to multiple ruptures around the optic disc area, visible as angioid streaks, followed by localized atrophy of RPE and thinning of the choriocapillaris [54]. Ingrowth of fibrovascular tissue from the choroid shows predilection for the site of angioid streaks. Likewise, optic disc drusen may be caused by ectopic calcification and have been shown histologically to contain a high amount of calcium [20, 55]. Angioid streaks occured in 20 % in a series of 100 patients [56] and in 12.9 % in another series of 255 patients [20]. Being manifested after the age of 20 years, the findings were positively correlated with age.

Angioid streaks usually are asymptomatic. Visual disturbances may occur if a streak is located under the macula, resulting in subretinal hemorrhages, but these hemorrhages often resolve spontaneously with no evidence of CNV. The most significant visual complication of angioid streaks is the development of CNV from breaks in Bruch’s membrane [57]. Given that hemorrhages can occur without CNV, it is important to verify the presence of CNV before considering treatment.

In a most recent study by Barteselli et al. [20] a total of 70 of 255 patients (27.5 %) showed at least 1 retinal alteration characteristic of PXE. Peau d’orange was the most common finding (19.6 %), followed by angioid streaks (12.9 %), pattern dystrophy-like changes (7.5 %), and optic disc drusen (2.0 %). Frequency of PXE-like fundus changes (especially peau d’orange and angioid streaks) was significantly higher in patients with TI (52.0 %) compared with those with TM (11.1 %; *P* <0.001) [20]. The frequency of PXE-like fundus alterations increased with age [20, 28]. In multivariate analyses PXE-like fundus changes were more common in patients with TI and were correlated with age, use of iron-chelating therapy, and history of splenectomy [20]. Previous studies on the ocular phenotype in patients with b-thalassemia have concentrated on the association with angioid streaks, as observed in patients with PXE, and vascular tortuosity. The other fundus features characteristic of PXE, such as peau d’orange, optic drusen and pattern dystrophy-like changes, are well described in these patients by Barteselli et al. [20], except comet tail lesion within the periphery.. In those patients with RPE degeneration, RPE mottling and visual acuity changes, serum iron levels, serum ferritin levels and number of blood transfusions received all were higher than in those without these changes [22]. Numerous studies report retinal pathologies in beta-thalassemia: Aksoy et al. [39] report 4/43 (9.3 %) children with TM who were identified with retinal pathologies. Taneja et al. [22] identified RPE changes in 21 (25 %) of 84 patients and Gartaganis et al. [28], in 11/29 (37.9 %). Gartaganis reported leopard skin appearance although the term “peau d’orange” has been used only recently by Bartesseli [20] for the description of specific fundus changes in thalassemia. The lack of consistent terminology to describe RPE alterations is obvious through the various cross-sectional observational studies. Similar lesions may be reported with different terms (RPE mottling vs “peau d’orange”) while the general term RPE degeneration may include both RPE mottling and pattern dystrophy-like changes.

The current therapy for hemoglobinopathies has significantly improved survival, and (unless it does not have an equally beneficial effect on elastic tissue), PXE complications are likely to be seen more frequent in the near future. The hemoglobinopathy-associated PXE has, therefore, a particular research interest and may contribute to the better understanding of inherited PXE. An annual funduscopic examination by an ophthalmologist beyond the second decade of life and a radiographic examination of the limbs to detect arterial calcifications beyond the third decade are also recommended. A close follow-up with funduscopy and fluorescein angiography is required in patients with angioid streaks and appropriate treatment applied when choroidal neovascularization develops, to prevent ocular hemorrhages and loss of visual acuity [18].

### NonePXE-like retinal abnormalities

Several studies have reported an association of increased vascular tortuosity with chronic anemia, especially in elderly patients with TM [38], in patients with a critical reduction of the hematocrit [58], or in correlation with Aspartate Aminotransferase (AST), Hb, and ferritin levels. Patients with increased retinal vascular tortuosity had significantly higher levels of ferritin and AST, and a higher rate of splenectomy. Moreover, splenectomy remains as a factor related to increased vascular tortuosity, which may be related to a greater thrombotic risk after splenectomy [20, 59]. Increased retinal vascular tortuosity was present in 16.9 % of patients, without a significant difference between TI and TM groups in the study by Barteselli et al. [20] and in 1/29 in another study by Gartaganis et al. [28]. The mean age of the patients in the study by Gartaganis et al. (2.0 +/− 10.4 years) was significantly lower than that of the study by Barteselli et al. (37+/−9 years), and included only b-thalassemia major (TM) patients, while that of Barteselli et al. included a substantially bigger number of patients comprised of both b-thalassemia major (TM) and b-thalassemia intermedia (TI) patients. Although Barteselli et al. failed to find a correlation between vascular tortuosity and age, (as described by Incorvaia et al. [38]), they attribute that to the lack of quantitative evaluation of the retinal vascular tortuosity. In their study, a qualitative evaluation by two retinal specialists was performed instead.

### Sickle thalassemia

In sickle thalassemia, angioid streaks have been encountered [60], and a frequency of 10 % was reported in a group of 58 cases, by Aesopos et al. [61]. In another study, by Fanny et al., out of the 18 patients studied, 13 (72.2 %) presented with sickle cell retinopathy [62]. Overall, retinopathy in beta thalassemia appears similar to sickle cell retinopathy.

### Desferrioxamine retinopathy

Desferrioxamine mesylate is an iron chelating agent used in the treatment of chronic iron overload in patients with thalassemia major and other hematologic conditions requiring regular blood transfusions [63]. Desferrioxamine ocular toxicity as experimental finding is well documented [64]. Ocular complications have been revised recently by Di Nicola et al. [65]. Complications are mainly associated with high doses of DFO in young patients and low ferritin levels. Ocular toxicity usually presents as night-blindness, blurred vision, decreased visual acuity, color vision impairment, or cataract formation. Regular evaluation by an ophthalmologist is therefore of paramount importance.

Ocular findings of desferrioxamine toxicity include the so called desferrioxamine retinopathy. Patients may present with decreased visual acuity, scotomas, nyctalopia, photopsias and metamorphopsias. Ocular findings in funduscopy vary and may include RPE opacification or loss of transparency, foveomacular, macular, paramacular, papillary or peripapillary and peripheral degeneration or combination of those, RPE pigment changes, optic disc edema and optic atrophy. In fluorescein angiography during the earliest stages, blocked fundus fluorescence in a patchy pattern is followed by late staining. This pattern is seen due to loss of outer retinal and RPE transparency before the onset of pigmentary changes. Angiography may also reveal mottled fluorescence in the early-phase due to pigment mottling with late hyperfluorescence or without late staining, mottled macular hyperfluorescence or optic disc hyperfluorescence. Electroretinography can also be helpful in detecting early retinopathy. Characteristic findings are the prolonged rod and cone implicit times or reduced scotopic and photopic a and b wave amplitudes. Electroculography may show reduced light-peak to dark trough (Arden ratio) or even extinguished response in widespread retinal involvement.

Another key examination for desferrioxamine retinopathy is fundus autofluoresence (FAF). The abnormal increased or decreased FAF is mostly bilateral, and is more extended than funduscopically visible alterations. Progressive retinal pigment epithelium (RPE) damage is accompanied by progressive FAF changes. Various phenotypic patterns of abnormal FAF have been identified. Fundus autofluorescence is a helpful, fast, and noninvasive tool for monitoring the status of the macula in patients at risk of desferrioxamine toxicity. It may be useful in the decision to discontinue or switch the therapy in cases of particular high risk for disease progression. The progressive nature of the RPE changes suggests an important role in the evolution of visual loss during long-term treatment with desferrioxamine.

According to the above mentioned, patients with visual symptoms on desferrioxamine therapy should undergo careful clinical evaluation. If blood–retinal barrier breakdown pre-exists, there is an increased risk of developing retinopathy. Late hyperfluorescence on fluorescein angiography seems to be a reliable indicator of active retinopathy. RPE pigment mottling does not resolve with drug discontinuation and is a less sensitive measure of disease activity. Electrophysiologic tests such as the ERG and EOG are usually confirmatory and indicate more widespread retinal dysfunction than may be implied by funduscopy alone. Desferrioxamine should be promptly discontinued in symptomatic patients without life-threatening iron overload because of the likelihood of further worsening. The decision to continue therapy in patients with active retinopathy and electrophysiologic evidence of disease must clearly weigh the increased risk of further visual loss against the morbidity of progressive systemic iron overload. It is hoped that newer formulations of desferrioxamine or newer chelating agents might be shown to have less ocular toxicity than desferrioxamine.

Macular and/or peripheral pigmentary degeneration are the most common changes described in DFO retinopathy. The earliest fundus changes described include subtle opacification or loss in transparency of the outer retina and retinal pigment epithelium (RPE). These changes precede the development of RPE mottling. Peripapillary, papillomacular, and paramacular patterns of RPE degeneration have also been reported [33, 66].

Ocular toxicity secondary to prolonged treatment with desferrioxamine may result in night blindness, centrocaecal scotoma, constriction of the peripheral visual field, pigmentary retinopathy, or optic neuropathy [29, 31, 67–69]. Treatment of thalassemia necessitates long-term blood transfusions with consequent iron overload of vital organs, for which chelation therapy is mandatory. Desferrioxamine toxicity is thought to arise secondary to chelation of metals such as iron, copper, and zinc, which are essential for normal retinal function [70, 71].

In a study on Desferrioxamine retinopathy by Viola et al. [35], authors detected a variety of pattern dystrophy-like changes or minimal changes affecting the RPE-Bruch membrane-photoreceptor complex. Multimodal imaging documented that fundus changes were more diverse and widespread than expected from slit lamp biomicroscopy alone. In concordance with previous histologic description of DFO retinopathy by Rahi et al. [32], multimodal imaging demonstrated that photoreceptor outer-derived retinoids, various fluorophores, and RPE displacement or clumping play an important role in DFO retinopathy. Most cases of pattern dystrophy-like changes end up in frank RPE atrophy.

Cases of rapid development of severe toxic retinopathy were associated with continuous intravenous desferrioxamine [72]. Frequent serum ferritin level monitoring and maintaining the desferrioxamine dosage with therapeutic index (daily dose per body weight (mg/kg) divided by serum ferritin level (mg/l)) below 0.025 [73], may safeguard against the development of rapidly progressive severe irreversible retinopathy. All patients who are on continuous intravenous desferrioxamine infusion, require close ophthalmologic monitoring, as prompt drug discontinuation might potentially arrest the retinal damage. It is important to follow up closely all patients on high dose therapy and to detect retinopathy in its earliest stages. Patients should be examined prior to therapy as subtle changes may otherwise escape or thought to be unrelated to medication. ERG and EOG may be diagnostic in cases of toxicity attributable to high-dose intravenous desferrioxamine.

## Value of retinal imaging

### Optical coherence tomography

Spectral domain OCT, which allows for detailed assessment of retinal microanatomy in vivo, has been used to study PXE-related retinal features [74] and to measure choroidal thickness in eyes affected by PXE using enhanced depth imaging-OCT. In a case report, Wu et al. [75], described the accumulation of multiple hyper-reflective deposits primarily in the choroid, retina pigment epithelium (RPE), and inner segment and outer segment (IS/OS) junction, in a 34 year old man with thalassemia major who complained of nyctalopia and decreased vision following high-dose intravenous deferoxamine. In a recent case control SD – OCT study of retinal and choroidal thickness, El-Shazly et al. [76] concluded that thalassemic patients can develop a significant decrease in foveal thickness. It seemed that foveal thickness is independently affected by the type of chelator. Deferoxamine affected foveal thickness more significantly than the oral chelator deferasirox. The lack of the effect of these medicines on the choroidal thickness points to a direct effect on the fovea and not the nutritive choroid layer.

### Fluorescein angiography

Despite the importance of novel techniques such as SD-OCT, fluorescein angiography remains the gold standard for detecting and documenting leakage from a CNV in patients with angioid streaks.

The fluorescein angiographic findings of angioid streaks, have been studied extensively in the past [77–79]. In the absence of other signs of CNV, however, fluorescein angiography does not usually add clinically relevant information and therefore may be refrained from in asymptomatic patients.

### Indocyanine green angiography

Indocyanine green angiography uses Near Infrared (NIR) light for excitation of the chromophore, which is superior to fluorescein angiography in detecting abnormalities under the RPE.

Indocyanine green is less well suited to detect leakage from CNV compared to fluorescein angiography. Due to its invasive nature, ICG is not recommended for monitoring patients. ICG is extremely useful in case of suspected Polypoidal Choroidal Vasculopathy (PCV), or occult CNV and to further investigate ocular pathophysiology in PXE-like changes in beta-thalassemia.

### Fundus autofluorescence

Fundus autofluorescence (FAF) has recently emerged as a novel noninvasive imaging technique that uses the fluorescent properties of innate fluorophores accumulated in the retinal pigment epithelium (RPE) to assess the health and viability of the RPE/photoreceptor complex.

Fundus AF imaging with most commonly used blue or green excitation light allows evaluation of the integrity and health of the RPE in vivo. Various phenotypic patterns of abnormal FAF can be identified with cSLO imaging. Fundus autofluorescence is a helpful, fast, and noninvasive tool for monitoring the status of the macula in patients at risk of DFO toxicity. It may be useful in the decision to discontinue or switch the therapy in cases of particular high risk for disease progression. The progressive alterations of the RPE, suggests an important role of pathologic RPE changes in the evolution of visual loss during long-term treatment with DFO [36].

Fundus AF reveals RPE atrophy as of hypo autofluorescent areas often more extensive than the areas of atrophy seen on funduscopy. Fundus AF is regarded useful as a non-invasive tool to monitor progression of RPE-like changes, including chorioretinal atrophy.

In DFO retinopathy multimodal imaging demonstrated that fundus changes were more diverse and widespread than expected by ophthalmoscopy. Consistently with previous histologic description of DFO retinopathy, multimodal imaging confirmed that photoreceptor outer-derived retinoids, various fluorophores, and RPE displacement or clumping are involved in DFO retinopathy, finally leading to frank RPE atrophy in most cases of pattern dystrophy-like changes. Follow-up examinations revealed progressive development of RPE atrophy in areas of pattern dystrophy-like changes [35].

### Confocal reflectance imaging

Confocal NIR reflectance imaging is highly sensitive in detecting peau d’orange and angioid streaks [74, 80]. The low absorption rate of 790 nm light by melanin within the RPE, in combination with the high contrast of a confocal imaging system, leads to the superior illustration of such structural alterations underneath the RPE cell layer. With NIR reflectance imaging, angioid streaks appear as uniform, well demarcated dark fissures against a lighter background, even when they remain unnoticed on color imaging.

## Functional alterations

### Visual acuity and visual fields

Night blindness and Visual field defects in patients on high-dose intravenous DFO, as well as visual loss due to optic neuropathy, defects in color vision and abnormal dark adaptation in patients receiving daily subcutaneous infusions of similar large doses of desferrioxamine for a prolonged period [19, 26, 29, 31, 40, 68] are the most frequently reported findings in relation to visual acuity and visual field testing.

### Electrophysiology

Electrophysiological techniques provide further insight regarding the pathophysiology of the retinal damage.

Electroretinographic and visual-evoked potentials (VEP) in thalassemic patients who had never received high doses of DFO showed abnormalities very similar to those reported in early siderosis bulbi. These included a b1-wave of significantly higher amplitude at 1 min and at the alpha point. VEPs showed a N1-P1 amplitude significantly greater than in controls, pointing to an important causative role of iron in their genesis [30]. Delayed visual evoked potentials have been detected after intravenous or subcutaneous infusion of large doses of DFO over a prolonged period of time [29]. ERG and EOG testing may indicate earlier or more widespread injury than is suggested by fundus examination alone [33].

## Current guidelines

Although there was a general consensus between available guidelines for the management of patients with TM, some guidelines proved to be more comprehensive and up to date than others. Minor differences regarded the optimal strategy for the assessment of iron overload and, notably, variations in the recommendations for iron chelation therapy. However, as the amount of new evident grows, the advances in the field gather, and our knowledge and understanding of the disease deepens, a final conclusive consensus seems achievable. Establishment of local guidelines in resource - poor countries is encouraged, as the availability and cost of novel diagnostic techniques and interventions poses an important limitation [81].

In beta-thalassemia, the need for regular blood transfusions, as well as the need for iron chelation treatment to reduce iron store of the body and prevent vital organ failure is well established. Therefore, future studies on beta-thalassemia treatment are most likely to compare efficacy and safety between different treatments without any control arm of no treatment. Studies with inadequate sample sizes are less informative than desired. The beta-thalassemia treatment literature regarding ocular findings also contains many non-randomized studies. Regardless of the underlying reasons, the fact that subjects were not drawn from the same population and randomized to one of a number of treatments means that it is again difficult to draw any meaningful conclusions. All of these problems could be overcome with more rigorous study design, which should include at least two groups, preferably randomized, and some kind of a priori sample size calculation to increase the likelihood of being able to answer the question motivating the study in the first place.

It is not true that only prospective randomized studies should be conducted but other study designs need to be purposefully designed to answer some question. For example, large observational studies or clinical data registries could be used to assess harms of treatment that might not be detected in smaller, randomized studies. Similarly, these non-randomized designs could also provide information about the incidence or prevalence of visual impairment or disability among various groups.

## Summary and future directions

In summary, patients suffering from β-thalassemia present with diverse ocular and systemic manifestations. Ocular findings range from decreased visual acuity, colour vision anomalies and nyctalopia, to visual field defects, cataract, retinopathy and optic neuropathy. Although iron-chelating agents, like desferrioxamine and deferriprone, are reported to be the cause of many of these ocular changes, most of the ocular changes in beta-thalassemia patients are attributed to the course and severity of the disease itself. Reduction in serum iron and ferritin levels by iron-chelating agents is the mainstay of current treatment for TM. Ophthalmic side-effects of these agents can be prevented, or at least controlled by regular ophthalmic examination, thus ameliorating any severe ocular complications.

### Limitations

This review has several limitations. Firstly, most observational studies or clinical data registries were small (only three included more than 100 patients).

Moreover, there is a risk bias that separate studies may have used the same sample source without being clearly identified. Secondly, some results were based on a limited number of studies and need to be proved by future studies. Finally, it is inescapable that the diversity of patients’ characteristics, such as age and physical conditions, or differences in beta-thalassemia treatment protocols in the past may confound in some degree the results. Overall, the most appropriate chelation regimen remains to be proved. Well designed, long-term RCTs are the only way forward.

## Conclusion

The selection of a suitable method for assessment and monitoring of fundus changes in beta-thalassemia constitutes an essential component of the design of clinical trials investigating efficacy and safety of iron chelators.

Advances in the assessment of change with imaging devices may shift the macroscopic standard of fundus photographs towards the microscopic and three-dimensional imaging of the retinal layers. With the increasing resolution and reliability of imaging technologies, it is likely that the estimation of these lesions and monitoring of change will be more precise and individualized in the near future. In addition, advances and implementation of image stabilization technologies should improve reproducibility of structural measurements to enable better differentiation of variability from true progression.

The awareness of subclinical disease as well as the identification of systemic factors associated with higher prevalence of beta-thalassemia retinopathy will aid the clinician in identifying those patients who are at higher risk of retinopathy. Yearly follow up ocular examinations are recommended in patients on iron chelators in the majority of guidelines for the management of patients with TM. However, more frequent examinations may be necessary in patients on high-dose intravenous DFO treatment. Newer imaging technologies (FAF, cSLO) allow for a precise estimation and close monitoring of fundus changes and could be integrated in trials assessing efficacy and safety of iron chelators.

## Abbreviations

BCVA, best corrected visual acuity; BUT, tear break-up time; CNV, choroidal neovascularization; cSLO, confocal scanning laser ophthalmoscope; DFO, desferrioxamine; EOG, electrooculographic; ERG, electroretinographic; FAF, fundus autofluorescence; Hb, hemoglobin; IDA, iron deficiency anemia; NIR, near infra red; PCV, polypoidal choroidal vasculopathy; PXE, pseudoxanthoma elasticum; RBC, red blood cell count; RPE, retinal pigment epithelium; RVT, retinal venous tortuosity; TI, thalassemia intermedia; TM, thalassemia major; VEP, visual-evoked potential
